# The Efficacy and Safety of Anlotinib in the Treatment of Thyroid Cancer: A Systematic Review

**DOI:** 10.3390/jcm14020338

**Published:** 2025-01-08

**Authors:** Calin Muntean, Adelaida Solomon, Remus Calin Cipaian, Razvan Constantin Vonica, Anca Butuca, Vasile Gaborean, Ionut Flaviu Faur, Catalin Vladut Ionut Feier

**Affiliations:** 1 Medical Informatics and Biostatistics, Department III-Functional Sciences, “Victor Babes” University of Medicine and Pharmacy Timisoara, 300041 Timisoara, Romania; cmuntean@umft.ro; 2 Clinical Department, Faculty of Medicine, “Lucian Blaga” University of Sibiu, 550169 Sibiu, Romania; calin.cipaian@ulbsibiu.ro; 3 County Clinical Emergency Hospital of Sibiu, 550245 Sibiu, Romania; 4 Preclinical Department, Faculty of Medicine, “Lucian Blaga” University of Sibiu, 550169 Sibiu, Romania; razvanconstantin.vonica@ulbsibiu.ro (R.C.V.); anca.butuca@ulbsibiu.ro (A.B.); 5 Department of Oncology, Elysee Hospital, 510040 Alba Iulia, Romania; 6 Department of Surgical Semiology, Faculty of Medicine, “Victor Babes” University of Medicine and Pharmacy Timisoara, 300041 Timisoara, Romania; vasile.gaborean@umft.ro; 7 IInd Surgery Clinic, Timisoara Emergency County Hospital, 300723 Timisoara, Romania; flaviu.faur@umft.ro; 8 X Department of General Surgery, “Victor Babes” University of Medicine and Pharmacy Timisoara, 300041 Timisoara, Romania; 9 Abdominal Surgery and Phlebology Research Center, “Victor Babes” University of Medicine and Pharmacy Timisoara, 300041 Timisoara, Romania; catalin.feier@umft.ro; 10 First Surgery Clinic, “Pius Brinzeu” Clinical Emergency Hospital, 300723 Timisoara, Romania

**Keywords:** Anlotinib, thyroid cancer, medullary thyroid carcinoma, differentiated thyroid cancer, systematic review, efficacy, safety

## Abstract

**Background and Objectives:** Anlotinib, a novel multi-kinase inhibitor targeting angiogenesis and tumor proliferation pathways, has shown promising efficacy in various cancers. Its role in treating thyroid cancer, particularly radioactive iodine-refractory differentiated thyroid cancer (RAIR-DTC), medullary thyroid carcinoma (MTC), and anaplastic thyroid carcinoma (ATC), is of significant clinical interest. This systematic review aims to evaluate the efficacy and safety of Anlotinib in patients with thyroid cancer, analyzing outcomes such as progression-free survival (PFS), overall survival (OS), response rates, and adverse events. **Methods:** A comprehensive literature search was conducted using PubMed, Scopus, and Web of Science databases up to October 2023. The review included randomized controlled trials and prospective studies assessing Anlotinib in thyroid cancer patients. Data extraction and quality assessment were performed independently by two reviewers following PRISMA guidelines. **Results:** Six studies involving a total of 277 patients were included. In patients with RAIR-DTC, Anlotinib demonstrated significant improvement in median PFS and objective response rates. In advanced or metastatic MTC, Anlotinib significantly prolonged median PFS compared to placebo, with high objective response rates. Subgroup analyses showed that older patients and those with bone metastases benefited significantly from Anlotinib treatment. In patients with ATC, Anlotinib-based chemotherapy yielded a 60% objective response rate. Anlotinib was also effective as neoadjuvant therapy in locally advanced thyroid cancer, achieving an objective response rate of 76.9%. Common adverse events included hypertension, proteinuria, and palmar–plantar erythrodysesthesia syndrome, which were generally manageable. **Conclusions:** Anlotinib appears to be an effective and well-tolerated treatment option for patients with various types of thyroid cancer, providing significant improvements in PFS and objective response rates. Further large-scale randomized studies are warranted to confirm these findings and to explore long-term outcomes.

## 1. Introduction

Thyroid cancer is the most common endocrine malignancy, with an increasing incidence worldwide over the past few decades [1,2]. It encompasses a heterogeneous group of tumors derived from thyroid follicular epithelial cells or parafollicular C cells [3]. Differentiated thyroid cancer (DTC), including papillary and follicular thyroid cancers, accounts for the majority of cases and generally has an excellent prognosis with a high survival rate [4,5]. However, a subset of patients with DTC become refractory to radioactive iodine (RAI) therapy, termed radioactive iodine-refractory differentiated thyroid cancer (RAIR-DTC), and exhibit poor outcomes [6,7]. Additionally, dedifferentiated high-grade differentiated thyroid carcinoma (DHGDTC) represents an aggressive variant of DTC that loses the ability to uptake iodine, further complicating treatment and worsening prognosis [8]. Anaplastic thyroid carcinoma (ATC) is another highly aggressive form characterized by rapid growth and early metastasis and is associated with a significantly lower survival rate compared to other thyroid cancer types [9]. Medullary thyroid carcinoma (MTC), arising from parafollicular C cells, represents approximately 2% of all thyroid cancers but contributes to a disproportionate number of thyroid cancer-related deaths due to its aggressive nature [10,11].

Treatment options for RAIR-DTC and advanced MTC are limited and pose significant clinical challenges [12,13,14,15,16]. Traditional therapies such as surgery and RAI are often ineffective in these settings. Surgery alone or systemic chemotherapy has shown limited efficacy and considerable toxicity, underscoring the need for novel therapeutic strategies [17,18]. The management of these advanced thyroid cancers necessitates the exploration of targeted therapies that can improve patient outcomes while maintaining an acceptable safety profile.

Angiogenesis plays a pivotal role in the progression and metastasis of thyroid cancers. Overexpression of vascular endothelial growth factor (VEGF) and its receptors has been observed in thyroid cancer cells, promoting tumor growth and angiogenesis [19]. This understanding has driven the development and clinical investigation of multi-kinase inhibitors (MKIs) that target these angiogenic pathways [20]. MKIs such as sorafenib and lenvatinib have been approved for the treatment of (RAIR-DTC, demonstrating improved progression-free survival. Additionally, cabozantinib has been established as a second-line treatment for RAIR-DTC. For RAIR-DTC patients harboring NTRK and RET fusions, specific inhibitors targeting these genetic alterations have been introduced, enhancing therapeutic precision. In MTC, selpercatinib and pralsetinib have shown efficacy by specifically targeting RET mutations. Furthermore, in the context of DHGDTC and ATC, treatment strategies are increasingly guided by molecular signatures. For instance, the combination of dabrafenib and trametinib is employed for BRAF-positive ATC, while a combination of immunotherapy with pembrolizumab and lenvatinib has demonstrated promising results in ATC management. Despite these advancements, challenges such as resistance, adverse side effects, and limited patient eligibility persist, underscoring the need for ongoing research and the development of additional therapeutic options for advanced thyroid cancers [21].

Anlotinib is a novel oral MKI that inhibits multiple targets, including VEGFR, PDGFR, FGFR, c-Kit, and Ret [22]. It has shown promising anti-tumor activity in various solid tumors, including lung cancer and soft tissue sarcoma. By inhibiting both tumor angiogenesis and proliferation pathways, Anlotinib presents a potential therapeutic advantage in the management of advanced thyroid cancers. Its favorable pharmacokinetic profile and manageable toxicity have spurred interest in evaluating its efficacy in thyroid cancer patients [22]. Recent clinical studies have investigated the role of Anlotinib in thyroid cancer and have demonstrated encouraging results, with significant improvements in progression-free survival and response rates in patients with advanced thyroid cancer [23]. Moreover, Anlotinib has been observed to induce early biochemical responses, as evidenced by reductions in serum thyroglobulin levels, and has been generally well tolerated among patients.

In light of these encouraging results, it is essential to thoroughly assess the existing evidence concerning the effectiveness and safety of Anlotinib in patients with thyroid cancer. This systematic review intends to compile and integrate data from current clinical studies to offer a comprehensive insight into Anlotinib’s therapeutic potential in this setting. By examining metrics such as progression-free survival, overall survival, response rates, and adverse events, the review aims to inform clinical practices and steer future research initiatives in the treatment of advanced thyroid cancers.

## 2. Materials and Methods

### 2.1. Criteria for Inclusion and Exclusion

This systematic review incorporated studies that fulfilled the following inclusion requirements: (1) clinical research involving individuals diagnosed with thyroid cancer types such as RAIR-DTC, MTC, or ATC, who received Anlotinib either as a standalone treatment or alongside other therapies; (2) publications detailing clinical outcomes including progression-free survival (PFS), overall survival (OS), objective response rate (ORR), disease control rate (DCR), biochemical responses, and safety data like adverse events (AEs); (3) randomized controlled trials (RCTs), prospective cohort studies, and phase II or III trials to ensure the inclusion of robust evidence; and (4) articles written in English to facilitate accurate interpretation and data extraction.

Studies were excluded based on the following criteria: (1) non-human research such as laboratory experiments or animal studies; (2) formats like case reports, case series, reviews, commentaries, and editorials that did not provide original data; (3) studies that did not offer adequate information on clinical outcomes or safety related to Anlotinib treatment; (4) research where Anlotinib was not the main intervention or its effects were confounded by other treatments; and (5) unpublished manuscripts or grey literature to ensure the reliability and quality of the data.

### 2.2. Sources of Information

A thorough search of the literature was performed using three major databases: PubMed, Scopus, and Web of Science. The search included all relevant publications up until October 2024 to capture the latest studies on Anlotinib in the context of thyroid cancer. These databases were selected for their extensive coverage of biomedical and clinical research. Additionally, the reference sections of pertinent articles were manually reviewed to identify any additional studies that met the inclusion criteria. The review protocol was registered on the Open Science Framework with the identifier osf.io/p7ma2.

### 2.3. Strategy for Literature Search

To ensure comprehensive coverage, the search strategy was meticulously crafted to identify all pertinent studies on Anlotinib and thyroid cancer. This involved using a combination of Medical Subject Headings (MeSH) and relevant keywords such as “Anlotinib”, “thyroid neoplasm”, “differentiated thyroid carcinoma”, “medullary thyroid cancer”, “anaplastic thyroid cancer”, “radioactive iodine-resistant”, “clinical trial”, “progression-free survival”, “overall survival”, “response rate”, and “adverse effects.” Boolean operators (AND, OR) were employed to refine the search effectively. For instance, the PubMed search query was structured as (“Anlotinib”) AND (“thyroid cancer” OR “differentiated thyroid carcinoma” OR “medullary thyroid cancer” OR “anaplastic thyroid cancer”) AND (“clinical trial” OR “progression-free survival” OR “response rate”).

### 2.4. Process for Selecting Studies

Two independent reviewers evaluated the titles and abstracts for eligibility based on the predefined inclusion and exclusion criteria. Full-text versions of potentially relevant studies were subsequently obtained and independently assessed by both reviewers. Any disagreements during the selection process were resolved through discussion or by consulting a third reviewer. The entire selection procedure was documented following the Preferred Reporting Items for Systematic Reviews and Meta-Analyses (PRISMA) guidelines to ensure clarity and reproducibility.

### 2.5. Extraction of Data and Variables

Data extraction was carried out independently by two reviewers using a standardized form to ensure consistency. The information gathered included details about the study (such as author, publication year, country, and design), participant demographics (including sample size, age, and gender), disease specifics (type of thyroid cancer, stage, and previous treatments), intervention parameters (Anlotinib dosage and duration of treatment), outcomes measured (PFS, OS, ORR, DCR, biochemical responses), and safety information (AEs and toxicity grades). Any discrepancies in data extraction were addressed through discussion. The methodological quality of the included studies was evaluated using appropriate instruments, such as the Cochrane Risk of Bias tool for randomized trials.

### 2.6. Assessment of Study Quality

The quality of each included study was appraised using the Newcastle-Ottawa Scale, which is tailored for observational studies. This tool examines aspects such as the selection of study groups, the comparability between groups, and the ascertainment of exposure and outcomes. Each study received a rating of low, moderate, or high risk of bias based on the scoring criteria. These quality assessments were crucial for interpreting the findings, with studies identified as having a high risk of bias being noted separately. Additionally, sensitivity analyses were planned to determine the influence of these higher-risk studies on the overall results.

## 3. Results

**Figure 1:**
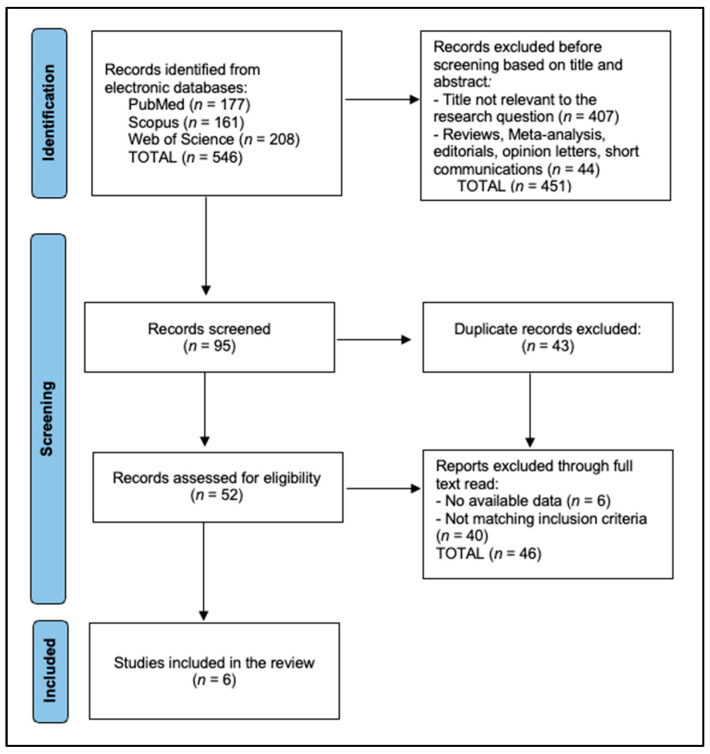
PRISMA flow diagram.

Table 1 provides an overview of the six studies [24,25,26,27,28,29] included in the final analysis. All studies were conducted in China between 2021 and 2024 and collectively involved a total of 277 participants (Figure 1). Of a total of 546 articles that were identified in the three databases, 451 were excluded before screening based on title and abstract. Additionally, 43 duplicates were further excluded, resulting in 52 records screened for eligibility, of which 6 were included in the study. The studies vary significantly in sample size, from smaller cohorts such as Sun et al. [24], with only 10 patients in a prospective cohort study, to Chi et al. [27], which included 113 participants (76 receiving Anlotinib and 37 on placebo) in a randomized controlled trial (RCT). Notably, Li et al. [25] had 91 participants in a Phase IIb RCT, and Zhao et al. [26] conducted a post hoc analysis on a subgroup of Li’s sample, adding depth to the findings. The single-arm studies by Zheng et al. [28] and Huang et al. [29] enrolled 25 and 13 patients, respectively, contributing moderate-quality evidence to the overall dataset.

The average age of participants ranged broadly, with Zheng et al. [28] reporting the oldest mean age of 65.0 ± 9.8 years, while Huang et al. [29] had the most diverse age range from 14 to 80 years. Gender distribution varied across studies, with a relatively balanced male-to-female ratio in some (e.g., Sun et al. [24] with 5 males and 5 females) and a more skewed distribution in others, such as Chi et al. [27] where the Anlotinib group had 33 males and 43 females.

The thyroid cancer type and prior treatments also differed significantly between studies. Patients had either radioiodine-refractory differentiated thyroid cancer (RAIR-DTC) or metastatic medullary thyroid cancer (MTC), with some studies, like Zheng et al. [28], focusing on anaplastic thyroid cancer (ATC). Treatment histories varied, with most patients receiving surgery (e.g., 89% in Li et al. [25] and 100% in Chi et al. [27]), while some studies reported high use of additional therapies like RAI therapy (e.g., 97% in Chi et al. [27]). Zhao et al. [26] focused on MTC patients with negative prognostic factors, underscoring the need to account for baseline disparities in treatment outcomes (Table 2).

Dosage protocols were consistent, with most studies administering 12 mg of Anlotinib daily on a 2-week on/1-week off schedule, except for Zheng et al. [28], who combined Anlotinib with chemotherapy in a regimen of 12 mg daily for 14 days within a 21-day cycle. Follow-up durations varied, from an early median assessment of 6 weeks in Sun et al. [24] to 35.9 months in Chi et al. [27], which was the longest follow-up period. Progression-free survival (PFS) was significantly improved in Anlotinib groups across studies, with Chi et al. [27] reporting a median PFS of 40.5 months for RAIR-DTC patients versus 8.4 months for the placebo group (*p* < 0.001). Similarly, Li et al. [25] observed a median PFS of 20.7 months for Anlotinib compared to 11.1 months for the placebo (*p* = 0.029), and Zheng et al. [28] reported a median PFS of 25.1 weeks in advanced anaplastic thyroid cancer (ATC) cases treated with Anlotinib and chemotherapy.

Objective response rate (ORR) and biochemical responses further highlighted Anlotinib’s efficacy. ORR was particularly notable in Chi et al. [27], where the Anlotinib group achieved an ORR of 59.2% compared to 0% in the placebo group. Similar efficacy was observed in other studies, with Li et al. [25] reporting an ORR of 48.4% in the Anlotinib group versus 3.4% for placebo and Zheng et al. [28] showing an ORR of 60% in ATC patients. Biochemical responses, such as a 72.8% reduction in thyroglobulin levels reported by Sun et al. [24], further supported Anlotinib’s role in disease control, particularly for RAIR-DTC. Zhao et al. [26] noted substantial benefits in older patients with poor prognostic factors, indicating Anlotinib’s potential in targeted patient subsets (Table 3).

Table 4 details the safety profiles and adverse events associated with Anlotinib across the studies, demonstrating a high incidence of treatment-related adverse events (TRAEs). Anlotinib was associated with a 100% incidence of TRAEs in several studies, including those by Sun et al. [24], Li et al. [25], and Chi et al. [27], although these events were generally manageable. Common TRAEs included hypertension, proteinuria, and palmar–plantar erythrodysesthesia (PPE) syndrome, with Grade ≥ 3 adverse events being reported in a substantial proportion of patients, particularly in Chi et al. [27] (76% of the Anlotinib group). Serious adverse events (SAEs) were also observed, with Li et al. [25] and Chi et al. [27] reporting 12.9% and 16% serious TRAEs, respectively, indicating a moderate risk profile in some patients.

The safety profile was particularly notable in specific patient subgroups. Zhao et al. [26] observed a higher incidence of Grade ≥ 3 TRAEs (55.6%) in older patients, which was similar to the broader patient population studied by Li et al. [25]. Zheng et al. [28] showed a lower incidence of Grade ≥ 3 adverse events (28%) in a smaller cohort, likely reflecting the differences in study design and population characteristics. Despite the high incidence of TRAEs, the studies concluded that the adverse effects were generally consistent with Anlotinib’s known profile and were manageable within a clinical setting (Table 4).

## 4. Discussion

### 4.1. Summary of Evidence

This systematic review evaluated the efficacy and safety of Anlotinib in patients with advanced thyroid cancer, including RAIR-DTC, MTC, and ATC. The findings from the included studies indicate that Anlotinib offers significant clinical benefits in terms of progression-free survival and objective response rates across different thyroid cancer subtypes. Moreover, in patients with RAIR-DTC, Anlotinib demonstrated substantial improvements in median PFS and ORR. Chi et al.’s randomized controlled trial reported a median PFS of 40.5 months in the Anlotinib group compared to 8.4 months in the placebo group, with an ORR of 59.2% [27]. These results are particularly noteworthy given the limited treatment options and poor prognosis associated with RAIR-DTC. Sun et al.’s study also showed early disease control and significant biochemical responses, suggesting that Anlotinib may be effective even in heavily pretreated patients [24].

In advanced MTC, Li et al.’s Phase IIb trial showed that Anlotinib significantly prolonged median PFS and increased ORR compared to placebo [25]. Zhao et al.’s subgroup analysis further demonstrated that older patients and those with bone metastases derived significant benefits from Anlotinib, highlighting its potential utility in high-risk patient populations [26]. Similarly, for patients with ATC, Zheng et al.’s study revealed that Anlotinib-based chemotherapy achieved an ORR of 60% and a median PFS of 25.1 weeks [28]. Considering the aggressive nature of ATC and the lack of effective treatments, these findings are promising and suggest that Anlotinib could be a valuable addition to the therapeutic arsenal for ATC.

Huang et al.’s study explored the use of Anlotinib in the neoadjuvant setting for locally advanced thyroid cancer [29]. The high ORR of 76.9% and a significant R0/R1 resection rate indicate that Anlotinib may facilitate surgical interventions by reducing tumor size and extent, potentially improving long-term outcomes. Regarding safety, while the incidence of treatment-related adverse events was high, these were consistent with the known side effects of angiogenesis inhibitors. Hypertension, proteinuria, and palmar–plantar erythrodysesthesia syndrome were the most common adverse events but were generally manageable with appropriate interventions such as dose adjustments, supportive care, and monitoring. Serious adverse events were relatively rare, and treatment discontinuation due to adverse events was infrequent.

Another similar study that was excluded from the final analysis for being a case report discussed a patient with primary squamous cell carcinoma of the thyroid, treated with Anlotinib combined with sintilimab [30]. The treatment led to a significant reduction in tumor volume observed over five courses, although it ultimately resulted in fatal liver complications after five months. This highlights the dual potential of combination therapies to both significantly impact tumor size and introduce severe side effects such as immune-related liver damage. Conversely, the study by Su et al. [31] focused on a patient with recurrent and metastatic radioactive iodine-refractory differentiated thyroid cancer harboring TERT promoter and BRAFV600E mutations. Here, Anlotinib was used as a sole agent after initial placebo control and demonstrated a remarkable 93.79% reduction in the target lesions over 52 cycles, coupled with progression-free survival of over 37 months. This extended control of disease underscores the efficacy of Anlotinib in RAIR-DTC, particularly in the context of specific genetic alterations.

In a similar manner, Liang et al. [32] presented a comprehensive analysis of Anlotinib’s role in inhibiting angiogenesis in anaplastic thyroid cancer, demonstrating its ability to suppress cell viability and hypoxia-activated angiogenesis both in vitro and in vivo. Crucially, their findings revealed that Anlotinib blocks the CXCL11-EGF-EGFR feedback loop, implicating the AKT-mTOR pathway as part of this interaction, thereby offering a novel insight into the mechanism by which Anlotinib could exert its effects on both cancer and endothelial cells under hypoxic conditions. Contrastingly, Zhang et al. [33] reported on the efficacy of Anlotinib combined with radioiodine in treating a rare case of scalp metastasis from papillary thyroid cancer, with significant tumor size reduction observed after just three cycles of neoadjuvant Anlotinib, followed by maintenance therapy post-thyroidectomy. This dual approach not only provided clinical benefits by reducing the need for more invasive procedures such as craniotomy but also highlighted the versatility of Anlotinib in managing metastatic and differentiated thyroid cancers, albeit in a significantly less aggressive form than ATC.

Peng et al. [34] reported on the visibly curative effects of dabrafenib and trametinib in two patients with advanced poorly differentiated thyroid carcinoma, noting significant symptom relief and tumor shrinkage. This underscores the potential of these targeted therapies in personalized oncological treatment. Conversely, Yu et al. [35] analyzed data from 1310 patients across seven studies, finding that all TKIs improved progression-free survival compared to placebo, with lenvatinib showing the greatest benefit. However, only apatinib and Anlotinib also significantly improved overall survival. This broader analysis suggests that while TKIs are effective in managing disease progression in radioiodine refractory differentiated thyroid cancer, their impact on overall survival and the incidence of severe adverse events varies, thereby guiding clinical decision-making on the optimal choice of TKI for RAIR-DTC.

A meta-analysis that included 25 studies highlighted the substantial benefits of targeted therapies like vandetanib, sorafenib, and lenvatinib in improving progression-free survival (PFS) and overall survival (OS) with hazard ratios of 0.35 and 0.53, respectively [36]. They also reported high objective response rates (ORRs) and disease control rates (DCR), notably emphasizing apatinib’s superior performance in terms of ORR. Conversely, Ji et al. [37] used a network meta-analysis to compare 13 treatments across 12 randomized controlled trials, affirming the significant PFS and OS benefits of targeted therapies, particularly pointing out lenvatinib as the most effective in PFS enhancement. Both studies underscore the potential of these therapies to substantially benefit patients with RR-DTC, albeit acknowledging the considerable adverse event profiles associated with these drugs, which necessitates careful patient selection and management. Together, these studies advocate for the use of targeted therapies in RR-DTC, providing critical data that assist in optimizing treatment strategies.

In a similar manner, the study by Nava et al. [38] found that the use of the multi-kinase inhibitor sorafenib resulted in a 70% reduction in tumor size in a 32-year-old patient with a 7.8 cm locally advanced papillary thyroid carcinoma, thereby enabling complete surgical resection after six months of therapy. This aligns with the findings of Yeo et al. [39], who reported that three out of four patients with T4a differentiated thyroid cancer experienced significant tumor reduction with tyrosine kinase inhibitor therapy, allowing for successful total thyroidectomy and subsequent radioactive iodine treatment. Both studies demonstrated the potential of kinase inhibitors as effective neoadjuvant treatments, facilitating surgical intervention in cases previously deemed unresectable. Additionally, Yeo et al. [39] highlighted a 100% disease-specific survival rate over 29 to 75 months, reinforcing the positive outcomes observed by Nava et al. [38]. These consistent results across different cohorts underscore the promising role of kinase inhibitors in managing locally advanced thyroid carcinomas, although the limited sample sizes suggest that further research is necessary to generalize these findings.

These collective findings suggest that Anlotinib is effective and generally well-tolerated across different thyroid cancer types and stages. Its ability to improve progression-free survival and induce significant tumor responses positions it as a promising therapeutic option in advanced thyroid cancers, where treatment choices are limited.

### 4.2. Limitations

In conclusion, Anlotinib appears to be an effective and generally well-tolerated treatment option for patients with advanced thyroid cancer, including RAIR-DTC, MTC, and ATC. The evidence indicates significant improvements in progression-free survival and objective response rates with manageable safety profiles. However, the differences between the included studies, such as variability in sample sizes, patient demographics, and study designs, pose a strong limitation for the generalizability of these conclusions. Despite these limitations, the findings support the consideration of Anlotinib as a therapeutic option in clinical practice, particularly for patients with limited treatment alternatives. Further large-scale, multicenter randomized trials are warranted to confirm these results, explore the long-term benefits and safety of Anlotinib in more diverse patient populations, and establish its role in combination with other therapies.

## 5. Conclusions

Anlotinib appears to be an effective and generally well-tolerated treatment option for patients with advanced thyroid cancer, including RAIR-DTC, MTC, and ATC. The evidence indicates significant improvements in progression-free survival and objective response rates with manageable safety profiles. These findings support the consideration of Anlotinib as a therapeutic option in clinical practice, particularly for patients with limited treatment alternatives. Further large-scale, multicenter randomized trials are warranted to confirm these results, to explore the long-term benefits and safety of Anlotinib in diverse patient populations, and to establish its role in combination with other therapies.

## Figures and Tables

**Table 1:** Characteristics of included studies.

Study and Author	Country	Year	Study Design	Sample Size	Study Quality
Sun et al. [24]	China	2024	Prospective Cohort Study	10	High
Li et al. [25]	China	2021	Randomized Controlled Trial (Phase IIb)	91	High
Zhao et al. [26]	China	2024	Post hoc Analysis of RCT	Subgroup of [25]	High
Chi et al. [27]	China	2023	Randomized Controlled Trial (Phase II)	113 (76 Anlotinib, 37 Placebo)	High
Zheng et al. [28]	China	2023	Single-Arm Study	25	Moderate
Huang et al. [29]	China	2022	Single-Arm Phase II Trial	13	Moderate

RCT—Randomized controlled trial.

**Table 2:** Patient demographics and baseline characteristics.

Study and Author	Sample Size	Mean Age (Years)	Gender (Male/Female)	Thyroid Cancer Type	Prior Treatments
Sun et al. [24]	10	55.8 ± 9.7	5/5	RAIR-DTC	Surgery, RAI therapy, MKIs (60% prior MKIs)
Li et al. [25]	91	Anlotinib: 51.8 ± 10.6	Anlotinib: 42/20	Advanced or Metastatic MTC	Surgery (89%), Radiotherapy (26%), Chemotherapy (17%)
		Placebo: 50.9 ± 11.1	Placebo: 18/11		
Zhao et al. [26]	Subgroup of [25]	≥50 years	-	MTC with Negative Prognostic Factors	Surgery, Radio-chemotherapy
Chi et al. [27]	113	Median: 56 (Anlotinib), 57 (Placebo)	Anlotinib: 33/43	Locally Advanced or Metastatic RAIR-DTC	Surgery (100%), RAI Therapy (97%), Radiotherapy (18%)
			Placebo: 8/29		
Zheng et al. [28]	25	65.0 ± 9.8	13/12	Locally Advanced or Metastatic ATC	No Prior Therapy (First-line Treatment)
Huang et al. [29]	13	14–80 (Range)	Not Specified	Locally Advanced Thyroid Cancer	-

RAIR-DTC: radioiodine-refractory differentiated thyroid cancer; MTC: medullary thyroid cancer; ATC: anaplastic thyroid cancer; RAI: radioactive iodine; MKIs: multi-kinase inhibitors.

**Table 3:** Treatment details and efficacy outcomes.

Study and Author	Anlotinib Dosage	Follow-Up Duration	PFS	ORR	Biochemical Response	Results
Sun et al. [24]	12 mg, 2 weeks, on/1 week off	Median 6 weeks (early assessment)	NR	PR: 20%, SD: 80%	Tg decreased by 72.8%	Anlotinib is well tolerated; early disease control is achieved.
Li et al. [25]	12 mg once daily, 2 weeks, on/1 week off	Median 25.8 months	20.7 months (Anlotinib) vs. 11.1 months (Placebo), *p* = 0.029	ORR: 48.4% (Anlotinib) vs. 3.4% (Placebo)	Calcitonin levels decreased significantly	Anlotinib improved PFS, with acceptable safety.
Zhao et al. [26]	Subgroup analysis of [25]	Extended follow-up	Older patients: PFS 17.5 vs. 6.8 months, HR = 0.31, *p* = 0.002	ORR in older patients: 47.2%	Calcitonin and CEA levels decreased	Significant benefits in older patients and those with bone metastases were observed.
Chi et al. [27]	12 mg once daily, 2 weeks, on/1 week off	Median follow-up: 35.9 months	40.5 months (Anlotinib) vs. 8.4 months (Placebo), *p* < 0.001	ORR: 59.2% (Anlotinib) vs. 0% (Placebo)	NR	Anlotinib showed significant PFS benefits in RAIR-DTC.
Zheng et al. [28]	12 mg daily, days 1–14 per 21 days, plus chemotherapy	Median follow-up: 52.6 weeks	Median PFS: 25.1 weeks	ORR: 60%	NR	Anlotinib-based chemo was effective in ATC.
Huang et al. [29]	12 mg daily, 2 weeks on/1 week off, 2–6 cycles	NR	NR	ORR: 76.9%	NR	Anlotinib was effective as a neoadjuvant in locally advanced thyroid cancer.

PFS: progression-free survival; ORR: objective response rate; PR: partial response; SD: stable disease; NR: not reported; Tg: thyroglobulin; HR: hazard ratio; CEA: carcinoembryonic antigen; RAIR-DTC: radioiodine-refractory differentiated thyroid cancer; ATC: anaplastic thyroid cancer.

**Table 4:** Safety profiles and adverse events.

Study and Author	Incidence of TRAEs (Anlotinib)	Grade ≥ 3 TRAEs	Most Common TRAEs	Serious TRAEs	Conclusions on Safety
Sun et al. [24]	100%	Grade 3 AEs: Hypertension only	Hypertension (100%), Proteinuria (70%)	NR	Anlotinib is generally well tolerated within the initial 6 weeks.
Li et al. [25]	100% (Anlotinib), 89.7% (Placebo)	Grade ≥ 3: 58.1% (Anlotinib)	PPE syndrome (62.9%), Proteinuria (61.3%), Hypertension (46.8%)	Serious TRAEs: 12.9%	Most AEs are manageable, with an acceptable safety profile.
Zhao et al. [26]	Similarly to [25] in subgroups	Grade ≥ 3: 55.6% (older patients)	Hypertension, PPE syndrome, Proteinuria	Serious TRAEs: 16.7% (older patients)	The safety profile was similar to the entire population.
Chi et al. [27]	100% (Anlotinib), 87% (Placebo)	Grade ≥ 3: 76% (Anlotinib)	Hypertension (84%), PPE syndrome (74%), Proteinuria (65%)	Serious TRAEs: 16%	AEs are acceptable and consistent with the known profile.
Zheng et al. [28]	56% had at least one AE	Grade ≥ 3: 28%	PPE syndrome (28%), Diarrhea (16%), Hypertension (12%)	NR	AEs are manageable and tolerable.
Huang et al. [29]	Common AEs: Hypertension (77%), Hypertriglyceridemia (69%), Proteinuria (54%), TSH increase (54%), HFS (39%)	Most AEs were grade 1 or 2	NR	NR	AEs are consistent with known profile and are manageable.

TRAEs: treatment-related adverse events; PPE syndrome: palmar–plantar erythrodysesthesia syndrome; HFS: hand–foot syndrome; NR—not reported.

## Data Availability

No new data were created or analyzed in this study.
